# Arbitrary polarization and rotation multiplexed metasurface hologram

**DOI:** 10.1515/nanoph-2025-0313

**Published:** 2025-09-16

**Authors:** Zonge Che, Yisheng Dong, Tiaoming Niu, Jining Li, Guanmao Zhang, Ziyin Ma, Shujie Liu, Jingwei Zhang, Zhonglei Mei

**Affiliations:** Institute of Optoelectronics and Electromagnetic Information, School of Information Science and Engineering, 12426 Lanzhou University, Lanzhou 730000, China; Center for Terahertz Waves, College of Precision Instrument and Optoelectronics Engineering, and the Key Laboratory of Optoelectronics Information and Technology (Ministry of Education), Tianjin University, Tianjin 300072, China; School of Precision Instruments and Optoelectronics Engineering, Tianjin University, Tianjin 300072, China

**Keywords:** metasurface hologram, polarization switchable, rotation multiplexing, amplitude and phase control

## Abstract

Metasurface holograms, characterized by their ultra-small thickness, high efficiency, and low loss, hold significant potential for applications in optical information storage, optical field manipulation, and security encryption. This paper proposed a polarization switchable and rotation multiplexing metasurface based on simultaneous amplitude and phase modulation. By precisely designing meta-atoms, the geometric parameters and orientation angle are engineered to independently control the phase and amplitude distributions of the metasurface. Utilizing a polarization switching strategy, incident light with different polarization angles generates distinct holograms. Additionally, the rotation-multiplexing mechanism further enhances information storage capacity by rotating the metasurface disk, thereby increasing the diversity and degrees of freedom in hologram. The results demonstrate that this approach enables precise optical field manipulation across multiple degrees of freedom, facilitating the reconstruction of multichannel holograms. This method provides a novel technological pathway for high-density optical storage, information encryption, and dynamic optical display.

## 1 Introduction

Metasurfaces are artificial planar metamaterials composed of subwavelength structures. Compared to traditional materials, they exhibit a unique ability to manipulate electromagnetic waves [1]. Owing to their ultrathin thickness [2], high efficiency [3], and broadband response [4], [5], metasurfaces have been widely employed for electromagnetic wavefront control. Furthermore, they can modulate multiple degrees of freedom for EM waves, including amplitude [6], phase [7], and polarization [8], enabling diverse functionalities. This versatility has facilitated their application in various fields, such as metalenses [9], [10], [11], orbital angular momentum (OAM) [12], [13], [14], holograms [15], [16], [17], vector beam generation [18], [19], [20], among others.

To enhance the information capacity of metasurfaces, various holographic multiplexing techniques have been proposed, including spatial multiplexing [21], [22], [23], polarization multiplexing [24], [25], [26], [27], frequency multiplexing [28], orbital angular momentum multiplexing [29], [30], [31], rotational multiplexing [32], wavelength multiplexing [33], [34], [35], among others. Polarization multiplexing enables the encoding of multiple independent holograms on a metasurface by controlling the polarization state of the incident light. In recent years, researchers have introduced various innovative polarization multiplexing approaches. For instance, Zhu et al. [36], developed a multifunctional full-space metasurface that integrates frequency and polarization multiplexing, generating five distinct holograms under different linear polarization states at specific frequencies. Ren et al. [37], implemented a three-channel near-field and far-field multicolor image display using a single-layer nonorthogonal polarization multiplexing metasurface. Zhang et al. [28], proposed a dual-frequency polarization multiplexing metasurface capable of achieving four-channel hologram. These advances demonstrate that polarization multiplexing effectively overcomes the capacity limitations of conventional hologram, offering new opportunities for high-density information storage and display. Recently, cascaded metasurface hologram has gained significant attention due to its ability to achieve complex optical functionalities by stacking or arranging multiple metasurface layers in series. Wei et al. [32], introduced in-plane rotation as an additional multiplexing dimension and proposed a machine learning-based iterative gradient optimization method, enabling the reconstruction of six distinct holograms in both single-layer and dual-layer cascaded metasurface configurations. Wang et al. [38], developed a bilayer hybrid metasurface device comprising a rotating radiation-type metasurface (RTM) and a nonrotating birefringent metasurface (BM). Using a gradient descent optimization inverse design approach, they achieved customizable three-dimensional metasurface hologram with rotation-driven dynamic switching in the microwave regime. Despite these advancements, cascaded metasurface designs face several challenges. The large interlayer spacing required in stacked configurations increases the overall volume, limiting their practicality in certain applications. Additionally, precise interlayer alignment is crucial for optimal performance, necessitating complex fabrication processes that elevate manufacturing costs. Moreover, the holographic reconstruction algorithms for cascaded metasurfaces are computationally intensive, making the optimization process increasingly challenging and resource-demanding.

Based on this, we have designed and demonstrated a metasurface hologram approach that integrates both rotation and polarization multiplexing through simultaneous amplitude and phase modulation. Since the amplitude modulation follows Malus’ law, distinct holograms can be switched by rotating the metasurface disk counterclockwisely. Unlike previous dynamically reconfigurable holograms that rely on dual-cascaded metasurfaces, our method achieves rotation- and polarization-switchable hologram using a single metasurface. This approach offers several advantages, including a more streamlined and efficient design, as well as simplified fabrication. In our framework, the incident polarization angle and the metasurface rotation angle serve as independent degrees of freedom for hologram switching, thereby reducing design complexity while enhancing information capacity. This innovative design significantly expands the functionality and storage capacity of holographic displays, with potential applications in information storage, data encryption, and security concealment.

## 2 Results and discussion

**Figure 1:**
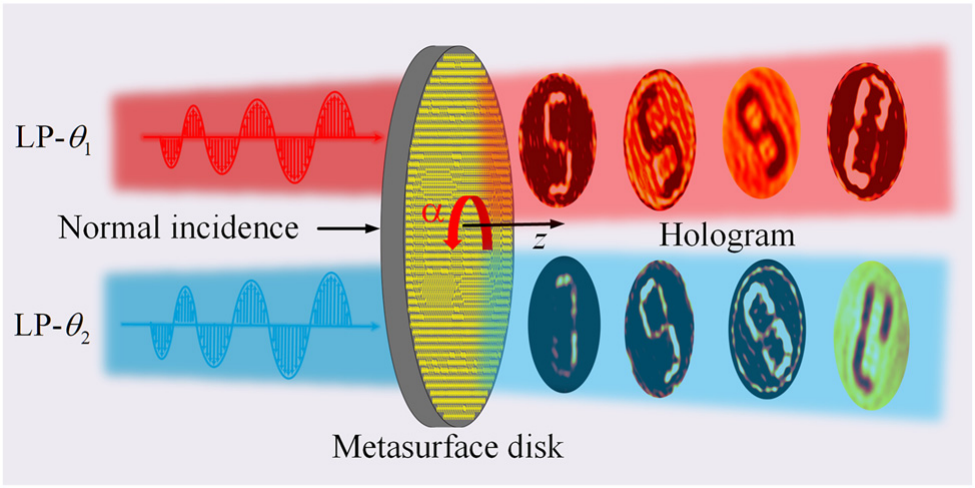
Schematic illustration of the designed metasurface hologram with polarization and rotation multiplexing.

As illustrated in Figure 1, we propose a novel design scheme for metasurface holograms that enables arbitrary polarization and rotational switching. The structure consists of a single-layer metallic meta-atom, where *α* denotes the counterclockwise rotation angle of the metasurface disk. When incident light with linear polarization angles *θ*_1_ and *θ*_2_ illuminates the metasurface, distinct holographic images are generated in the transmission space. In Figure 1, the rotation angles of the metasurface disk are set to *α* = 0°, *α* = 30°, *α* = 40°, and *α* = 160°. For a fixed *α*, different holograms emerge at varying incident polarization angles, demonstrating the functionality of polarization-controlled switching. Conversely, at a constant incident polarization angle, modifying *α* results in the formation of different holograms, thereby achieving rotational multiplexing.

### 2.1 Amplitude modulation principle

For the copolarized transmission intensity, the meta-atom can be approximated as an ideal polarizer for both the long- and short-axis resonance modes [39]. By setting *t*_*xx*_ = 1 and *t*_*yy*_ = 0, the transmitted intensity *I* can be formulated as:

(1) $$I=I_{0}\cos^{2}\left(\beta -\theta \right)$$

The detailed derivation of Equation (1) is provided in (detailed analysis is given in Note S1 (Supporting information)). Since the polarization angle *θ* can be arbitrarily selected, the above analysis confirms that the transmitted intensity can be modulated by adjusting the linear polarization (LP) direction while keeping the orientation angle *β* fixed. Consequently, *θ* functions as a switching parameter for channel coding.

### 2.2 Design of meta-atom

**Figure 2:**
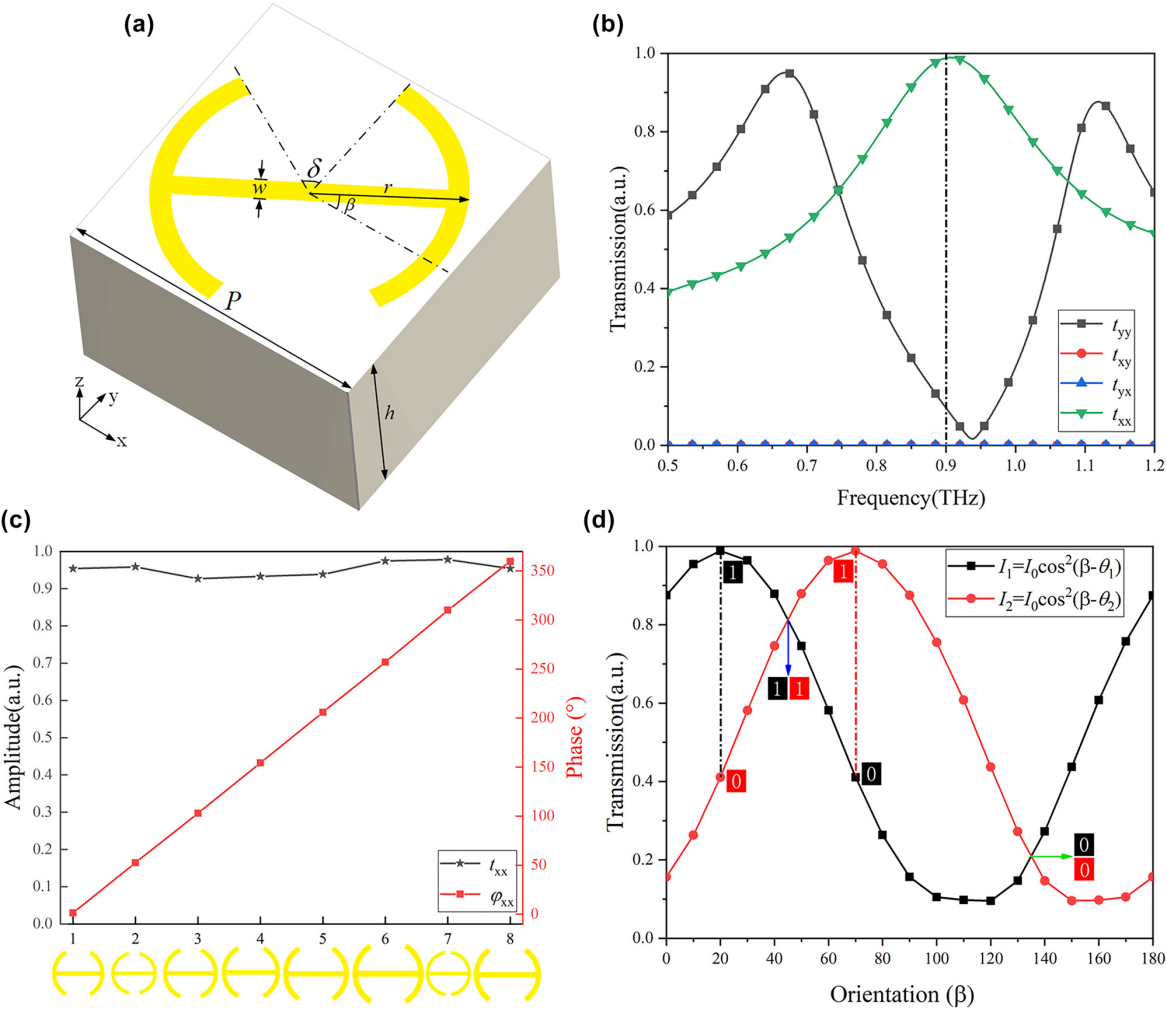
Characteristics and display principles of the meta-atom. (a) Three-dimensional structure of the meta-atom. (b) Transmission spectrum of the meta-atom. (c) Phase and amplitude characteristics of the eight selected double-split metal patterns at 0.9 THz. (d) Transmission intensity variation as a function of *β* under different incident polarization angles (*θ*_1_ = 20° and *θ*_2_ = 70°).

To achieve polarization-switchable functionality, we designed a meta-atom consisting of a silicon substrate and a double-split metal pattern, as illustrated in Figure 2(a). The period *P* = 80 μm, *h* represents the thickness of the silicon substrate. The metal pattern has a thickness of 0.3 μm and a width of *w* = 5 μm. The radius of the double-split ring and its opening angle are denoted as *r* and *δ*, respectively. The orientation angle of the metal pattern relative to the *x*-axis is given by *β*. Figure 2(b) presents the transmission spectrum of the x- and y-polarized components for the meta-atom. The simulation results reveal that under LP illumination along the *x*-axis, the designed meta-atom achieves maximum x-polarized transmission at approximately 0.9 THz, with an efficiency exceeding 95 %. This confirms that the double-slit metal pattern exhibits strong copolarized transmission for incident polarization components aligned with its long axis. To achieve phase modulation of the metasurface, the phase distribution is controlled by adjusting the geometric parameters of the meta-atoms. Specifically, the radius (*r*) and the opening angle (*δ*) of the double-split ring are primarily tuned, which in turn modulates the phase delay of the transmitted wave. By adjusting the geometric parameters *δ* and *r*, we selected eight distinct meta-atoms that satisfy the phase modulation requirements. Figure 2(c) illustrates the phase modulation characteristics of the metal pattern at the operating frequency of 0.9 THz. The results indicate that these eight meta-atoms achieve 0 − 360° phase shift with minimal variation in amplitude. Based on these meta-atoms, a metasurface can be constructed. Figure 2(d) depicts the amplitude modulation principle of the metasurface hologram, which operates through polarization angle switching. The black and red curves represent the relationship between transmission intensity and orientation angle at two different polarization angles *θ*_1_ and *θ*_2_. The simulated cosine-squared curve highlights the significant variation in transmission intensity for the same orientation angle under different polarization angles. In channel 1 (black curve *I*_1_), two different orientation angles *β* correspond to the same transmission intensity, while in channel 2 (red curve *I*_2_), the corresponding transmission intensity alternates between high and low values. Similarly, for channel 2, the orientation angle that yields the same transmission intensity corresponds to high and low values in channel 1. For the eight meta-atoms satisfying phase modulation, the transmission intensity curves as a function of *β* under polarization angles *θ*_1_ and *θ*_2_ are further discussed in Note S2 (Supporting information).

To evaluate the robustness of the proposed metasurface against fabrication imperfections, we conducted a tolerance analysis by introducing variations of ±1 μm in the key geometric parameters (and ±1° for angular parameters) from their nominal values. This range exceeds the typical fabrication accuracy of UV lithography in the THz regime (∼±0.2 μm), thereby providing a more conservative performance assessment. The variations in transmission amplitude and phase for different geometric parameters under both polarization states are discussed in Note S3 (Supporting information).

### 2.3 Polarization switchable rotation multiplexed metasurface design

**Figure 3:**
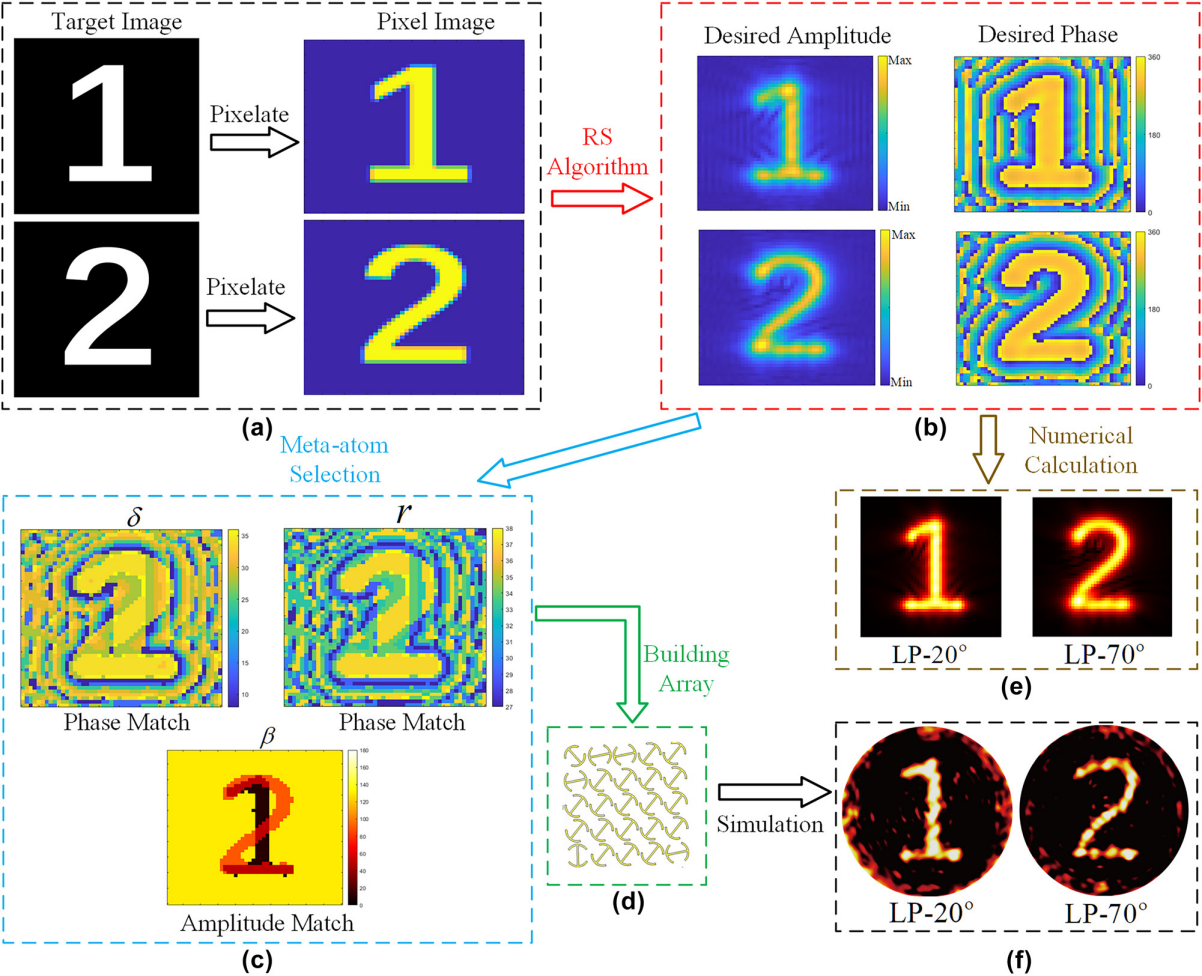
Schematic design of the dual-channel metasurface hologram. (a) Pixelation processing of the target image. (b) Calculation of the amplitude and phase distribution of the target image. (c) Match meta-atom parameters based on the desired amplitude and phase distribution. (d) Partial structure of the designed metasurface. (e) Numerically computed holographic reconstruction results. (f) Simulated holographic reconstruction results.

Referring to Figure 2, we arbitrarily take *θ*_1_ = 20° and *θ*_2_ = 70° to implement polarization switchable metasurface holograms. The design process for achieving this functionality is illustrated in Figure 3. As shown in Figure 3(a), the target image is first processed into a pixelated image using binarization techniques. The amplitude and phase distributions of the pixelated image are then computed based on Rayleigh–Sommerfeld diffraction theory, which can be expressed as:

(2) $$U_{1}\left(x_{1},y_{1}\right)=\frac{1}{j\lambda }\underset{S_{2}}{\iint }U_{2}\left(x_{2},y_{2}\right)\cos<n,r>\frac{\exp\left(jkr\right)}{r}\;dS_{2}$$

where *U*_1_(*x*_1_, *y*_1_) and *U*_2_(*x*_2_, *y*_2_) are the optical field distribution on the source plane and the imaging plane, respectively. $r=\sqrt{\left[{\left(x_{1}-x_{2}\right)}^{2}+{\left(y_{1}-y_{2}\right)}^{2}+{\left(z_{1}-z_{2}\right)}^{2}\right]}$ represents the distance between the point (*x*_1_, *y*_1_, *z*_1_) on the source plane (metasurface) and the point (*x*_2_, *y*_2_, *z*_2_) on the imaging plane. *λ* and *k* are the operating wavelength and the wave number in free space, respectively. Here, the pixelated image in Figure 3(a) represents the electric field intensity distribution *U*_2_(*x*_2_, *y*_2_) on the imaging plane. Based on Equation (2), the required electric field distribution *U*_1_(*x*_1_, *y*_1_) on the source plane (metasurface) is obtained through integral calculation, from which the complex amplitude and phase profiles of images “1” and “2” are computed, as shown in Figure 3(b). According to the reversibility of light propagation, if the electric field distribution *U*_1_(*x*_1_, *y*_1_) on the metasurface is known, the reconstructed field distribution *U*_2_(*x*_2_, *y*_2_) on the imaging plane can be obtained using the following formula [40]:

(3) $$U_{2}^{\prime }\left(x_{2},y_{2}\right)=\frac{1}{j\lambda }\underset{S_{1}}{\iint }U_{1}\left(x_{1},y_{1}\right)\cos<n,r>\frac{\exp\left(-ikr\right)}{r}dS_{1}$$

Based on the above equation, the theoretically calculated electric intensity distribution on imaging plane is shown in Figure 3(e), allows verification of whether the amplitude and phase of the target image are accurately reproduced.

To achieve phase matching, the calculated phase distributions of both images are first superimposed and then aligned with the eighth-order phase gradient presented in Figure 2(c). This process determines the spatial distributions of the meta-atom parameters *r* and *δ*, as illustrated in Figure 3(c). For amplitude matching, the calculated distributions for digit “1” and “2” are first obtained, then they are used to give a two-bit coded magnitude profile, where “00” and “11” refer to the dark and bright regions for both digits, and “01” and “10” represent the illuminated region by one digit. Finally, this coded magnitude map is used to find the *β* distributions for each meta-atom, where the coding scheme is clearly indicated in Figure 2(d). The final *β* distribution is depicted in Figure 3(c). By integrating both amplitude and phase matching, the meta-atom distribution for each pixel is precisely determined. Figure 3(d) provides a portion of the designed metasurface disk structure, which consists of a 50 × 50 array of meta-atoms. The distance between the source plane and the imaging plane is set to 200 μm. To validate the proposed design, full-wave electromagnetic simulations were conducted using CST. The simulation results are presented in Figure 3(f). Compared to the numerical calculation results shown in Figure 3(e), the simulation results further validate the accuracy and effectiveness of the polarization switchable hologram design. To evaluate the performance of the proposed dual-channel metasurface hologram, we calculated the working efficiency of each channel under its corresponding incident polarization. The efficiency is defined as the ratio of the integrated power of the reconstructed image within the target region to the total incident power [41], [42], [43]. The simulated reconstruction efficiencies in Figure 3(f) are 62.41 % for the LP-70° channel and 69.03 % for the LP-20° channel. Both channels exhibit high holographic efficiencies, and the small efficiency difference (approximately 7%) indicates balanced amplitude responses and low crosstalk between the channels, which demonstrates the robustness of our design.

In addition, the holographic reconstruction capability across different operational frequencies was examined to characterize the working bandwidth. Note S4 (Supporting information) shows the reconstructed images of “1” at several frequency points, indicating an effective operational bandwidth of approximately 0.78–1.02 THz.

**Figure 4:**
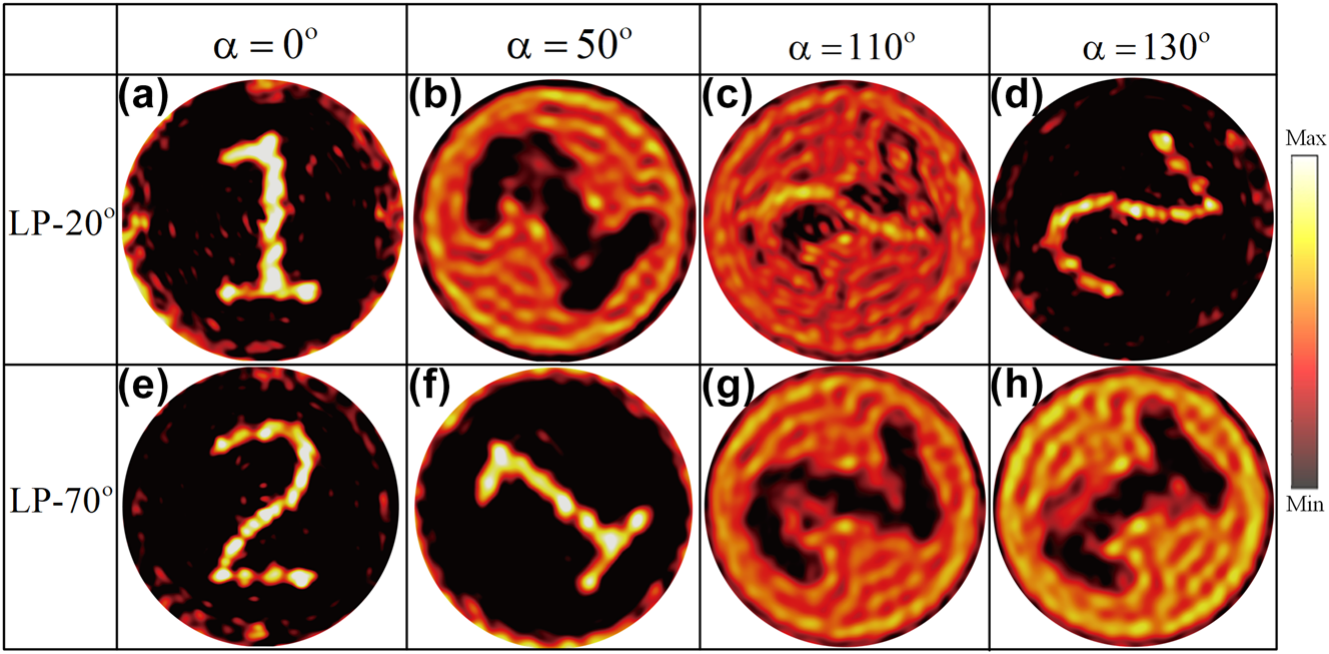
Simulated holographic imaging results for disk rotation angles of 0°, 50°, 110°, and 130° under an incident polarization angle of (a)–(d) *θ*_1_ = 20° and (e)–(h) *θ*_2_ = 70°.

**Figure 5:**
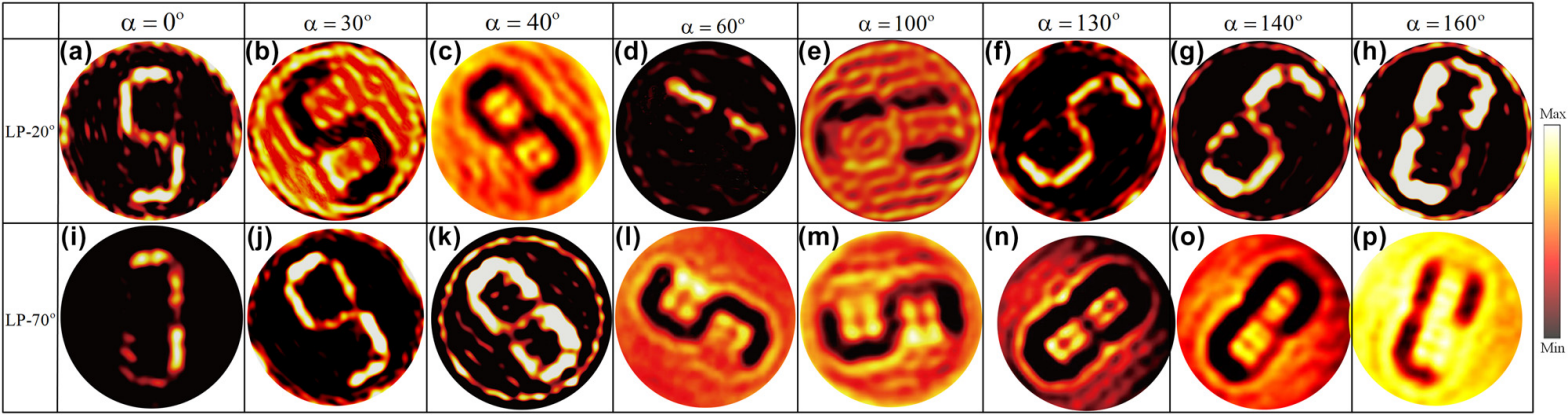
Simulated holographic imaging results for disk rotation angles of 0°, 30°, 40°, 60°, 100°, 130°, 140°, and 160° under an incident polarization angle of (a)–(h) *θ*_1_ = 20° and (i)–(p) *θ*_2_ = 70°.

Upon rotating the metasurface disk counterclockwise by an arbitrary angle *α*, the corresponding simulation results are presented in Figure 4. When the disk remains stationary (*α* = 0°), the metasurface generates high-quality holographic images of “1” and “2” under incidence angles *θ*_1_ = 20° and *θ*_2_ = 70°, respectively, with minimal background noise. As the disk rotates counterclockwise by 50°, the orientation angle distribution of each meta-atom changes, resulting in amplitude modulation effects. When the incidence angle is *θ*_1_, due to coupling errors between phase and amplitude, the background noise intensity exceeds that of the imaging region, degrading the quality of holographic reconstruction. In contrast, at an incidence angle of *θ*_2_, the imaging region exhibits higher intensity than the background noise, leading to the formation of a high-quality holographic image “1.” Similarly, when the disk rotation angle *α* reaches 110°, the increased background noise significantly degrades the hologram quality. At *α* = 130°, the holographic image of “2” appears under the incidence angle *θ*_1_. These results strongly demonstrate the feasibility of a polarization-switchable and rotation multiplexed metasurface hologram. However, it is important to note that the holograms “1” and “2” appearing during the disk’s rotation are predesigned, limiting the system’s ability to generate arbitrary new holograms. To enhance the information capacity of the metasurface disk, the disk must generate distinct holograms as it rotates. Consequently, we have designed a seven-segment digital tube image. The design procedure follows that presented in Figure 3, with the only distinction being the amplitude matching. In this case, the orientation angle *β* of each segment is different. After determining the meta-atom distribution for each pixel, a metasurface array consisting of 50 × 50 meta-atoms is constructed. Using 10° as the minimum rotation step, simulations were performed with arbitrary rotation of the metasurface disk. The corresponding results are shown in Figure 5. It is observed that when the disk’s rotation angle *α* is fixed, different incident angles *θ*_1_ and *θ*_2_ interacting with the metasurface produce distinct information. This property can be leveraged for optical storage or encryption, enabling information decoding based on the incident angle. Furthermore, as the disk rotates to different angles *α*, it can display distinct information under the same incident conditions. This functionality makes it suitable for applications in reconfigurable optical imaging and display systems, where different content can be observed from varying perspectives. The above analysis primarily considers two specific incident polarization angles *θ*_1_ and *θ*_2_. To extend its applicability, we have also simulated the metasurface response for other polarization angles. For further details, please refer to Note S6 (Supporting information).

### 2.4 Demonstration of metasurface hologram

**Figure 6:**
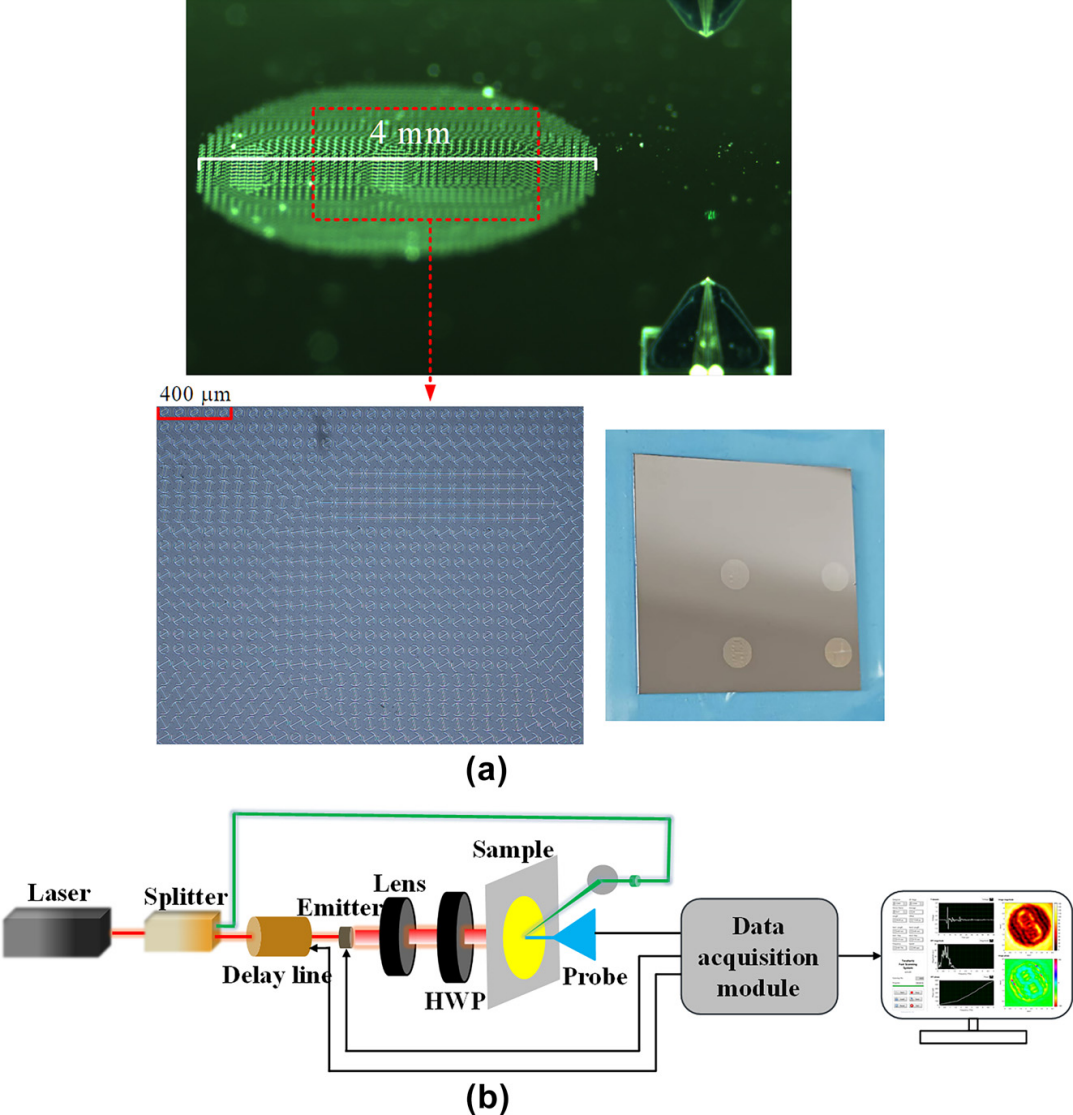
Fabricated metasurface sample and experimental characterization setup. (a) Microscopic image of the fabricated metasurface sample. (b) Schematic diagram of the NSTM system.

**Figure 7:**
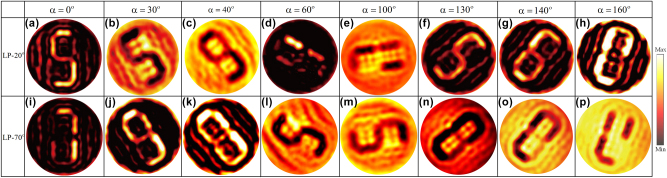
Measured near-field holographic images for disk rotation angles of 0°, 30°, 40°, 60°, 100°, 130°, 140°, and 160° under an incident polarization angle of (a)–(h) *θ*_1_ = 20° and (i)–(p) *θ*_2_ = 70°.

To experimentally validate the simulation results, a metasurface sample was fabricated using ultraviolet lithography, as shown in Figure 6(a). The fabricated metasurface was characterized using a near-field scanning terahertz microscopy (NSTM) system, as illustrated in Figure 6(b). In this setup, a THz near-field probe was utilized to measure the electric field distribution of the transmitted light. To enable precise two-dimensional scanning, the sample was mounted on a motorized translation stage. The probe was positioned 200 μm away from the metasurface, with a scanning range of 4.4 mm × 4.4 mm and a step interval of 0.1 mm. The experimentally obtained hologram at 0.9 THz, presented in Figure 7, exhibits excellent agreement with the simulation results, further validating the effectiveness of the designed metasurface.

In holographic imaging, the intensity distribution within the reconstruction region plays a crucial role in determining the overall image quality. Therefore, a series of quantitative evaluation methods are employed to assess the quality of the holographic reconstruction. To quantitatively evaluate the accuracy of image reconstruction, we utilize two commonly used metrics: Peak Signal-to-Noise Ratio (PSNR) and Structural Similarity Index (SSIM), as detailed below:

(4) $$PSNR=10\cdot \log_{10}\left(\frac{{MAX}_{\text{I}}^{2}}{MSE}\right)$$

where MAX_I_ denotes the maximum intensity (pixel value) in the holographic image. $MSE=\frac{1}{mn}\sum_{i=1}^{m}\sum_{j=1}^{n}{\left[I\left(i,j\right)-K\left(i,j\right)\right]}^{2}$ represents the mean squared error between the reconstructed holographic image and the target image. *m* and *n* represent the resolution of the image, *I* denotes the reconstructed holographic image, and *K* denotes the target image.

(5) $$SSIM\left(x,y\right)=\frac{\left(2\mu_{x}\mu_{y}+C_{1}\right)\left(2\sigma_{xy}+C_{2}\right)}{\left(\mu_{x}^{2}+\mu_{y}^{2}+C_{1}\right)\left(\sigma_{x}^{2}+\sigma_{y}^{2}+C_{2}\right)}$$

*μ* and *σ* denote the mean and standard deviation of the image, respectively, and *C*_1_ and *C*_2_ are stability constants used to prevent division by zero.

Table 1 provides a quantitative comparison between the simulated and experimental results. A noticeable degradation is observed in the experimental metrics compared to the simulations, which is primarily attributed to system noise and experimental uncertainties encountered during the measurement process. Despite this, SSIM analysis confirms that the proposed metasurface holograms are capable of reconstructing distinct target images with high fidelity. Interestingly, at certain rotation angles of the metasurface, the PSNR drops below 10 dB due to elevated background noise; however, the SSIM values remain relatively high, indicating that the structural similarity between the reconstructed and target images is well preserved.

**Table 1:** PSNR and SSIM comparison between Simulation and Measurement.

Method	*α* (deg)	PSNR (Sim) (dB)	PSNR (Meas) (dB)	SSIM (Sim)	SSIM (Meas)
LP-20°	0	17.22	13.60	0.79	0.72
	30	8.31	7.25	0.80	0.79
	40	7.13	6.46	0.82	0.78
	60	16.15	12.14	0.85	0.77
	100	7.26	6.55	0.77	0.75
	130	18.01	11.15	0.84	0.73
	140	18.33	12.33	0.88	0.74
	160	17.83	11.09	0.86	0.71
LP-70°	0	17.92	13.25	0.82	0.73
	30	18.20	13.31	0.87	0.78
	40	16.15	11.26	0.89	0.77
	60	9.05	8.17	0.85	0.79
	100	7.32	7.06	0.81	0.75
	130	17.98	13.43	0.83	0.79
	140	7.05	6.43	0.82	0.78
	160	6.62	6.35	0.86	0.81

## 3 Conclusions

The proposed holography method, based on amplitude and phase modulation for polarization- and rotation-multiplexed metasurface, successfully achieves multi-degree-of-freedom light field control, significantly enhancing both the resolution and information storage capacity of holographic imaging. The results demonstrate that this approach enables stable multichannel holographic reconstruction under arbitrary incident polarization angles and metasurface disk rotations. Compared with reconfigurable schemes based on phase-change materials, MEMS, or bilayer structures usually require external stimuli such as heating, electrical bias, or mechanical actuation, which increases fabrication complexity and may introduce additional loss. In contrast, our design remains structurally static and relies solely on the incident polarization angle and metasurface rotation as independent switching degrees of freedom. This enables ultrafast optical control (via polarization) combined with versatile state expansion (via rotation), without the need for additional integrated components. Consequently, our approach offers a low-cost, robust, and flexible platform for multichannel holographic switching. In the future, it can be further explored for applications in high-performance optical displays, information encryption, and optical computing, opening new research directions and application prospects for the advancement of metasurface-based optical technologies.

## Supplementary Material

Supplementary Material Details
