# Supplementary material for: Arbitrary polarization and rotation multiplexed metasurface hologram
Source: Nanophotonics. 2025 Sep 16;14(20):3307–16. doi: 10.1515/nanoph-2025-0313 (PMC12588565; doi:10.1515/nanoph-2025-0313)
Supplement: Supplementary file 1 — Supplementary Material Details [file j_nanoph-2025-0313_suppl_001.pdf]

Supporting Information

Arbitrary Polarization and Rotation Multiplexed Metasurface Hologram

*Zonge Che[1], Yisheng Dong[2], Tiaoming Niu[1], Jining Li[3], Guanmao Zhang[1], Ziyin Ma[1], Shujie Liu[1], Jingwei Zhang[1] and Zhonglei Mei[1]**

[1]Institute of Optoelectronics and Electromagnetic Information, School of Information Science and Engineering, Lanzhou University, Lanzhou 730000, China

[2]Center for Terahertz Waves, College of Precision Instrument and Optoelectronics Engineering, and the Key Laboratory of Optoelectronics Information and Technology (Ministry of Education), Tianjin University, Tianjin 300072, China

[3]School of Precision Instruments and Optoelectronics Engineering, Tianjin University, Tianjin 300072, China

E-mail: meizl@lzu.edu.cn (Zhonglei Mei)

These authors contributed equally to this work

Note S1

Amplitude modulation principle

Assuming that β represents the orientation angle of meta-atom, the transmitted light can be characterized by the corresponding Jones matrix:

$$J = R(-\beta)TR(\beta) = \begin{bmatrix} \cos\beta & -\sin\beta \\ \sin\beta & \cos\beta \end{bmatrix} \begin{bmatrix} t_{xx} & 0 \\ 0 & t_{yy} \end{bmatrix} \begin{bmatrix} \cos\beta & \sin\beta \\ -\sin\beta & \cos\beta \end{bmatrix} \quad \text{(S1)}$$

Where $R(\beta)$ is the rotation matrix, T is the transmission matrix. Here, t_{xx} and t_{yy} denote the co-polarized transmission coefficients of meta-atom along its long and short axes, respectively.

When a linearly polarized beam with a polarization angle θ is normally incident on the meta-atom, the Jone matrix of the transmitted beam can be written as:

$$J_{meta} = J \begin{bmatrix} \cos\theta \\ \sin\theta \end{bmatrix} = \begin{bmatrix} t_{xx}\cos(\beta-\theta)\cos(\beta) + t_{yy}\sin(\beta-\theta)\sin\beta \\ t_{xx}\cos(\beta-\theta)\sin(\beta) - t_{yy}\sin(\beta-\theta)\cos\beta \end{bmatrix} \quad \text{(S2)}$$

Given an incident beam intensity of I_0, the transmitted light intensity I can be expressed as:

$$I = I_0\left[t_{xx}^2\cos^2(\beta-\theta) + t_{yy}^2\sin^2(\beta-\theta)\right] \quad \text{(S3)}$$

By setting $t_{xx} = 1$ and $t_{yy} = 0$, the transmitted intensity I can be formulated as:

$$I = I_0\cos^2(\beta-\theta) \quad \text{(S4)}$$

Note S2

Transmission intensity response of the eight meta-atoms

As shown in Figure S1, for each meta-atom, when the orientation angle β varies within the range of $0° - 180°$, while parameters r and δ remain constant, the co-polarized transmission amplitude follows Equation (1) in the main text. This approach enables convenient control of the amplitude without disturbing the phase distribution, as long as the phase shift remains unchanged. Such a method significantly simplifies the design complexity.

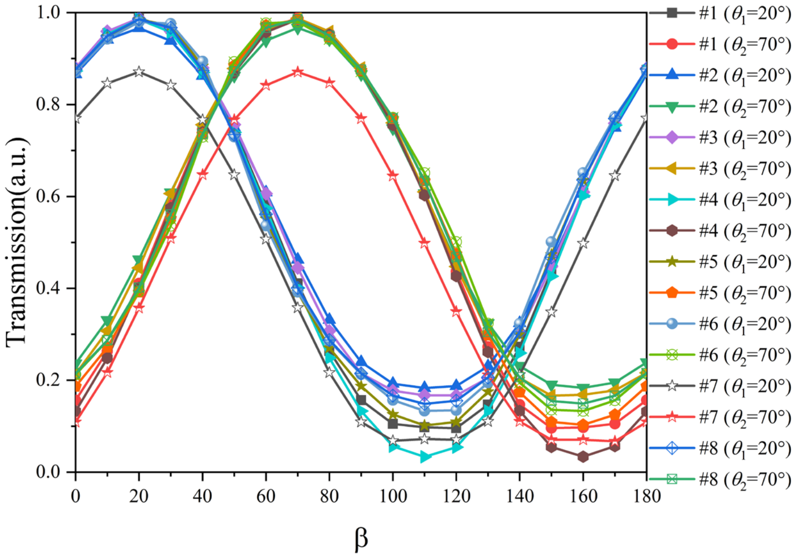

Figure S1. The transmission intensity variation curves corresponding to eight meta-atoms as β changes.

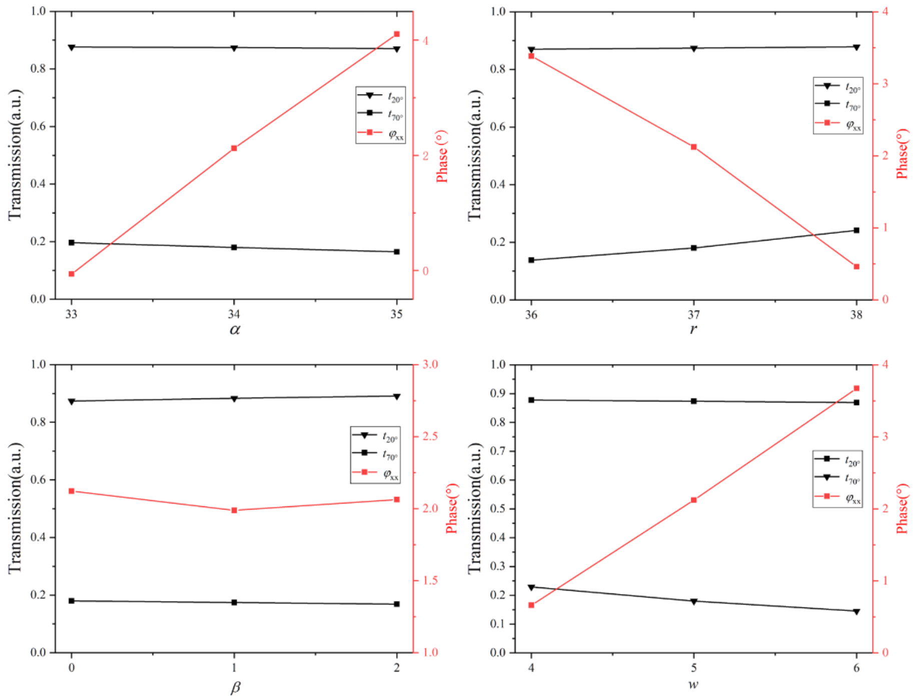

Figure S2. The transmission and phase variations corresponding to different geometric parameters

Note S3

Impact of fabrication tolerances on metasurface performance

As illustrated in Figure S2, meta-atom 1 in Fig. 2(c) is taken as a representative example. The results indicate that when α, r, and w are varied by $\pm1^{\circ}$, ±1 μm and ±1 μm, respectively, the phase experiences a shift of approximately $\pm2^{\circ}$, while the transmittance for the 20° polarization changes by about ±0.05, and that for the 70° polarization changes by about ±0.1. When β is varied by $\pm1^{\circ}$, the induced phase variation remains within $\pm0.2^{\circ}$, and the transmittance for both polarization states changes by approximately ±0.05. These findings confirm that the proposed metasurface preserves stable performance even in the presence of relatively large fabrication deviations.

Note S4

Holographic imaging results at different frequencies

It can be observed that the reconstructed image exhibits the highest fidelity at the central design frequency of 0.9 THz, while slightly blurred holograms are still observed at 0.78 THz and 1.02 THz. When the frequency further detunes to 0.77 THz or 1.03 THz, recognizable holographic images cannot be achieved.

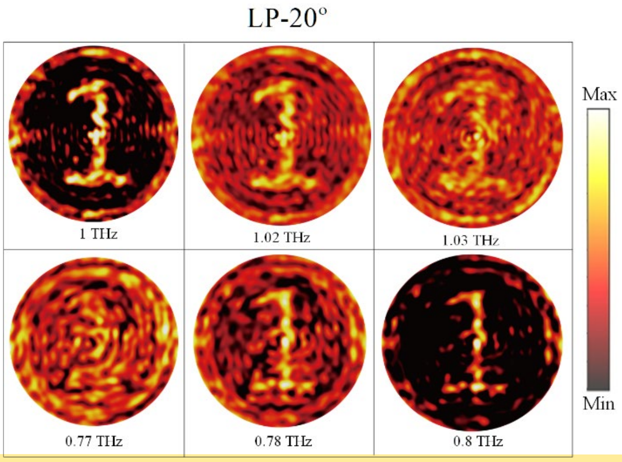

Figure S3. Holographic reconstruction at various frequencies.

Note S5

Amplitude and phase distributions of the target image

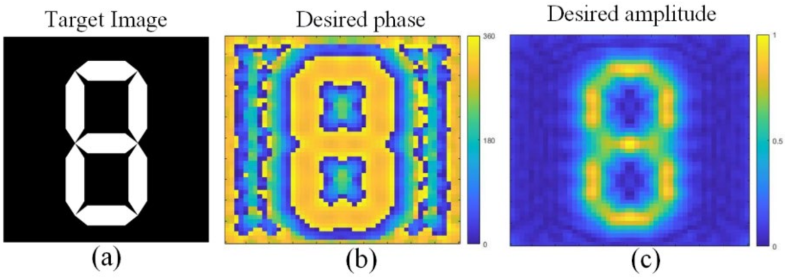

Figure S4. (a) Target image of a seven-segment tube. (b) Calculated phase distribution of the target image. (c) Calculated amplitude distribution of the target image.

The target image of the seven-segment digital tube display is shown in Figure S3(a). The desired phase and amplitude distributions are calculated using Equation (2) in the main text, as illustrated in Figure S3(b) and (c).

Note S6

Simulated holographic imaging results under other polarization angles of incidence

When the rotation angle of the metasurface disk is set to $0°$, the simulated holographic imaging results under various linearly polarized incident angles are shown in Figure S4. It can be observed that different incident polarization angles can generate either distinct or identical holographic images. When the metasurface disk is rotated, the resulting holographic images exhibit behavior similar to that described in the main text.

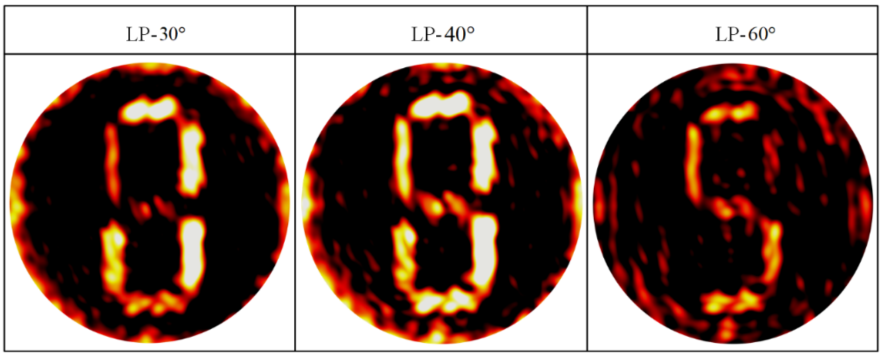

Figure S5. The hologram imaging results of the metasurface disk under arbitrary linear polarization angle incidence.
